# Editorial: Psychosocial repercussions of the Covid-19 pandemic for people living with or supporting others with diabetes

**DOI:** 10.3389/fcdhc.2022.1074162

**Published:** 2022-11-18

**Authors:** Andreia S. Mocan, Emma Berry, Mark Davies, Lene Eide Joensen, Rossella Messina

**Affiliations:** ^1^ Center for Diabetes, Nutrition and Metabolic Diseases, Emergency Clinical County Hospital, Cluj-Napoca, Cluj, Romania; ^2^ School of Psychology, Queen’s University Belfast, Belfast, Ireland; ^3^ Belfast City Hospital, Clinical Psychology Department, Northern Ireland, United Kingdom; ^4^ Steno Diabetes Center, Health Promotion Research, Gentofte, Denmark; ^5^ Department of Biomedical and Neuromotor Sciences, Alma Mater Studiorum-University of Bologna, Bologna, Italy

**Keywords:** diabetes, emotional distress, depression, anxiety, COVID-19

The outbreak of the Coronavirus (COVID-19) pandemic has created a global health crisis and resulted in drastic changes in the way we perceive our world. Imposed restrictions such as lockdown and social distancing, and the promotion of safety behaviours were introduced to reduce the magnitude of infection (1). In-person appointments were suspended and people were encouraged to manage their diabetes largely by themselves often without the immediate support of the health care team. Both for people with diabetes and for the health care team, new problems emerged. In addition to the usual challenges of diabetes self-management, emotional stress and adaptation, the COVID pandemic added a larger and more inclusive problem: adaptation to a new world. A new world that incorporates a new type of social and work environment and relationships; new rules, habits and behaviours; new perceptions and emotions related to self-protection and protection of others against a new and unknown threat. Within this context, this special issue, entitled “Psychosocial repercussions of the COVID-19 pandemic for people living with or supporting others with diabetes”, focussing on COVID-19 impact on diabetes was conceived.

This Research Topic attracted thought-provoking and insightful manuscripts that fall into 4 categories: a) COVID-19 and healthcare providers; b) COVID-19 emotional burden in type 1 diabetes (T1D); c) self-care activities and diabetes distress in type 2 diabetes (T2D) and d) general aspects of diabetes management during the COVID-19 pandemic.

The first category of research includes papers on professionals working in the field of diabetes. Hale et al. captures the concerns expressed by endocrine specialists and primary care providers about the well-being, psychosocial functioning, self-care routine, and financial challenges faced by the people living with diabetes (PWD) they care for. Lifestyle management interventions *via* tele medicine were used by the participating healthcare providers to offer support. Moreover, some of the participants helped PWD access financial programs when needed. A cross-sectional study by Wagner et al. found that half of the participating health care providers (n=123, 27 countries) experienced moderate to severe levels of mental stress, burnout, or other mental health issues because of the pandemic. Significant concerns were expressed about the lack of clear public health guidelines, delays in or the lack of COVID-19 testing, COVID-19 work exposure, safety concerns for themselves/staff/PWD, and about how PWD use technology and manage their diabetes. Isolation from colleagues was reported to be a moderate to serious problem for many participants in this study.

A second category of research examined people living with T1D. A literature review by Bassi et al. showed that the majority of COVID-19 studies on T1D teenagers focused mostly on glycemic control. Protective or risk factors, specific prevention or/and supporting methods for psychological well-being were less likely to be considered. ‘Positive lockdown effect’ was discussed and was defined as a period of stable routine had a positive influence on glycemic control. Caregivers perceived increased burden in diabetes management and child-related worry during the pandemic. Telemedicine was suggested as a method to increase maintenance of healthy diabetes care and self-efficacy during pandemic. An ecological momentary assessment study by Schmid et al., spanning a 10-day period, showed no difference between pre-pandemic and during pandemic levels of diabetes distress and depression among middle-aged individuals living with T1D. Fears of COVID-19 and of becoming infected were associated with diabetes duration and were predicted by pre-pandemic diabetes distress and illness/diabetes acceptance. O’Donnell et al. investigated the psychosocial and diabetes management impact of COVID-19 on adolescents living with T1D and who had elevated diabetes distress. They found that social and family relationships, health practices, school routines, diabetes management behaviors, dietary behaviors, physical activity and medical appointments were all disrupted. Resilience, social support, faith and meaning making were all reported as being helpful and protective against pandemic impact.

A third category of research focused on people living with T2D. A Scoping Review by Cummings et al. showed that the pandemic was in some ways detrimental to people living with T2D. Decreases in physical activity and no significant change in glucose monitoring and substance misuse were reported. However, positive effects were also found. Based on cross-sectional study of 1822 participants, Amerson et al. reported that during COVID-19 pandemic people living with T2D reported an increase in self-care activities, reductions in perceived distress and moderately lower diabetes distress, especially in vulnerable groups.

The fourth category of research concentrated on the impact pandemic lockdowns had on diabetes management in general. Olesen et al. found in a one year follow-up study that PWD reported both negative changes (i.e. lower physical activity, eaten unhealthier, diabetes management was more difficult; higher blood pressure and more glucose variability) and positive changes (i.e. more physical active, more healthy food and easier diabetes management) compared to pre-pandemic life. Moreover, the authors found that higher diabetes distress at the beginning of the pandemic was a predictor for both positive and negative aspects of diabetes management. Holloway et al. showed that PWD were feeling burned out by the constant effort to manage their diabetes during the pandemic and that easing/relaxing the pandemic restrictions might help with diabetes specific emotional problems such as worry about COVID-19 and diabetes.

For the members of the medical profession, the pandemic presented particular challenges about how to provide support to PWD to assist them to adapt whilst, at the same time, having to adapt themselves. Studies such as those published in this Special Issue, help to transform an unknown problem into a known one. Data derived from sound methodology, supports the development of conceptual frameworks upon which interventions to help PWD and healthcare professionals manage emotional struggles can be built. It should be noted that the studies presented in this issue tended to under-represent the views of men and of PWD from low and middle-income countries, where the experience of pandemic might be very different to those reported here. Nevertheless, the published studies are an important contribution to the understanding of the COVID-19 psychosocial and management impact on PWD.

## Author contributions

AM, EB, MD, LJ, and RM contributed to the conception of the editorial. AM redacted the draft of the present editorial All authors contributed to manuscript revision, read, and approved the submitted version.

## Conflict of interest

The authors declare that the research was conducted in the absence of any commercial or financial relationships that could be construed as a potential conflict of interest.

## Publisher’s note

All claims expressed in this article are solely those of the authors and do not necessarily represent those of their affiliated organizations, or those of the publisher, the editors and the reviewers. Any product that may be evaluated in this article, or claim that may be made by its manufacturer, is not guaranteed or endorsed by the publisher.
